# Multi-parametric speckle tracking analyses to characterize cardiac amyloidosis: a comparative study of systolic left ventricular longitudinal myocardial mechanics

**DOI:** 10.1007/s00380-022-02047-6

**Published:** 2022-03-31

**Authors:** Nikola Bogunovic, Martin Farr, Lukas Pirl, Cornelia Piper, Volker Rudolph, Fabian Roder

**Affiliations:** 1 Clinic for General and Interventional Cardiology/Angiology, Herz-und Diabeteszentrum Nordrhein-Westfalen, Ruhr Universität Bochum, Georgstraße 11, 32545 Bad Oeynhausen, NRW Germany; 2 Institut Für Röntgendiagnostik und Nuklearmedizin, Klinikum Braunschweig, Braunschweig, Germany

**Keywords:** Cardiac amyloidosis, Strain, Strain rate, Pre-systolic stretch index, Post-systolic index, Time-to-peak strain, Longitudinal displacement

## Abstract

Cardiac amyloidosis (CAM), the most common cardiac storage disease is associated with significant changes in left-ventricular (LV) morphology and function. To gain particular insights into LV systolic longitudinal myocardial mechanics we investigated seven parameters derived by speckle-tracking-echocardiography (STE) in patients with confirmed CAM (*n* = 59). The results were compared with those of individuals with healthy heart (*n* = 150) and another primary myocardial disease with also thickened myocardium and severe diastolic and systolic LV-dysfunction (symptomatic LV-non-compaction-cardiomyopathy, LV-NC, *n* = 30). In addition to standard echocardiographical measures, the STE-derived data were evaluated and documented utilizing polar-diagrams to obtain overviews of longitudinal myocardial mechanics of the entire LV. Compared with healthy individuals, patients with CAM and LV-NC showed significantly reduced LV-ejection-fraction (EF), global longitudinal systolic peak-strain, strain-rate, and displacement. Pre-systolic stretch-index, post-systolic index, and the EF/global peak-longitudinal-strain-ratio (EF/S) were increased. In contrast to healthy-hearts and the LV-NC group only patients with CAM demonstrated significantly reduced time-to-peak systolic longitudinal strain and time-to-peak strain-rate. Although the level of the segmental values in longitudinal mechanics was significantly different between the groups, comparable intraventricular baso-apical parameter-gradients were found for systolic longitudinal peak-strain and strain-rate, pre-systolic-stretch-index, post-systolic-index, and peak systolic displacement. Compared to ATTR-amyloidosis (ATTR-CAM), patients with AL-amyloidosis (AL-CAM) demonstrated significantly lower end-diastolic and end-systolic LV-volumes, LV-mass-indices, relative apical strain, time-to-peak systolic longitudinal strain, and time-to-peak longitudinal strain-rate. CAM and LV-NC demonstrated altered myocardial mechanics with significantly different STE-derived echocardiographical parameters. ATTR-amyloidosis and AL-amyloidosis had at least significantly different time-to-peak strain, time-to-peak strain-rate and relative apical sparing values.

## Introduction

Cardiac amyloidosis is the most frequent cardiac storage disease, which generates myocardial stiffness secondary to intramyocardial deposition of amyloidal fibrils with increasing severity and varying progression rates of diastolic and systolic LV-dysfunction over time according to the extent of myocardial infiltration, CAM-type [1–7], and other determinants [8–13].

Currently, especially AL-CAM is mostly verified using myocardial biopsy for histological and immune-histological analyses. Nuclear medicine offers tracers to identify ATTR-CAM [14–18]. In magnetic resonance imaging (MRI) intramyocardial diffuse late gadolinium enhancement (LGE) is often present [12] and the distribution of LGE may help to differentiate ATTR- and AL-CAM.

However, echocardiography is the preferred imaging technique at first patient contact that offers parameters which are highly suggestive of myocardial storage disease [19–24]. In this context, increased relative apical longitudinal strain (“apical sparing”) seems to be accepted as strong indicator for the presence of CAM [24–26]. Additional STE-derived parameters describing myocardial mechanics are of particular interest to potentially facilitate earlier diagnosis and to discriminate CAM from other myocardial diseases [21, 24] with thickened myocardium in combination with severe systolic and diastolic LV-dysfunction. However, to the best of our knowledge, no comprehensive dataset of STE-derived longitudinal segmental and global parameters with mappings of the myocardial mechanics of the entire LV exists for CAM [15, 20, 21].

## Aim of the study

Accordingly, the objective of the present study was to characterize longitudinal myocardial mechanics [19, 20] in patients with CAM. We compared the results with those of healthy hearts and another primary myocardial disease with thickened myocardium, increased LV mass and severe systolic and diastolic LV-dysfunction. Although hypertrophic cardiomyopathy (HCM) is more often in the differential diagnosis of CAM, both systolic and diastolic dysfunction are not universally present in patients suffering from HCM and comparison of strain analyses in CAM versus HCM is reported before [25]. Therefore, we used the data from a previous study conducted at our institution [21] of patients with symptomatic isolated LV-NC. The patients with LV-NC complied with the preconditions (i.e. thickened myocardium, increased LV mass, severe systolic and diastolic LV-dysfunction).

Our hypothesis was that it may be possible to characterize longitudinal myocardial mechanics in CAM—beyond relative apical strain alone—using different STE-derived parameters in comparison with the data for LV-NC and healthy hearts. Additionally, it should be investigated whether a differentiation between ATTR-CAM and AL-CAM could become feasible.

## Methods

### Study design

The present retrospective study was conducted between January and December 2020. Besides standard echocardiographical parameters, mappings of the longitudinal myocardial mechanics of the entire LV were created for each of the STE-derived parameters and groups using a 17-segment polar diagram [23]. All parameters in individuals with CAM were compared with those with LV-NC and normal hearts from a previous study conducted at our institution [21]. Additionally, significant differences between values in AL-CAM and ATTR-CAM were evaluated.

STE analyses included the following parameters:

1. Peak systolic longitudinal strain (%): change of myocardial length in the baso-apical direction compared with the initial value;
2. Peak systolic longitudinal strain rate (%/s): strain over time;
3. Time-to-peak longitudinal strain (ms): time from R-wave (ECG) to peak systolic strain;
4. Time-to-peak longitudinal strain rate (ms): time from R-wave to peak systolic strain rate.
5. Pre-systolic stretch index (%): early systolic stretching relative to the total combined shortening = sum of systolic stretching and shortening. Formula: pre-systolic stretch index = 100 × peak positive strain in early systole/(peak positive strain in early systole–peak negative strain in systole);
6. Post-systolic index (PSI) (%): relative amount of total shortening after aortic valve closure (AVC). Formula: PSI = 100 × (peak systolic strain after AVC–strain at AVC)/peak systolic strain after AVC;
7. Peak systolic longitudinal displacement (mm): systolic myocardial contraction movement in the baso-apical direction;

### Subjects

59 adult Caucasian patients (males/females 46/13, mean age 72.5 ± 10.2, range 38–89 years) with histo-pathologically confirmed CAM by endomyocardial biopsy were divided into two subgroups: ATTR-CAM (29 patients, males/females 27/2, mean age 79.2 ± 6.6, range 61–89 years) and AL-CAM (30 patients, males/females 19/11, mean age 66.0 ± 8.7, range 38–79 years, AL-lambda *n* = 28, AL-kappa *n* = 2). The echocardiogram used for the study was performed at first patient contact and no patient had medical treatment for amyloidosis before.

Patients with a QRS-width in the ECG > 120 ms (e.g. bundle branch blocks, pacemaker induced blocks) were not accepted to exclude any possible impact of this rhythm disorder on STE-derived analyses.

We compared the data in CAM with 30 patients with symptomatic isolated LV-NC (males/females 17/13, mean age 49.4 ± 17.4, range 21–81 years) [21]. The control group included 150 carefully selected individuals with healthy heart (75 males, 75 females, mean age 33.8 ± 11.5, range 16–76 years) who presented a normal ECG, normal exercise tolerance, normal clinical findings, and a normal echocardiogram [21].

### Echocardiography

Examinations were performed using a commercially available equipment (Vivid E9, GE, Horten, Norway) following the guidelines of the European Association of Cardiovascular Imaging and American Society of Echocardiography [32]. Myocardial LV imaging included the entire endo- and epicardium in all three apical standard views (i.e. 2-, 4-chamber, apical long axis). The images had to be accepted from the algorithm of the detection software (EchoPac, version 203, GE, Horten/Norway) taking the complete myocardial width from endo- to epicardium into account to prepare correct semi-automatic STE analyses. Patients with sinus rhythm were analyzed using three heart-beats, those with atrial fibrillation with at least three beats of medium scale R–R intervals.

A 17-segment polar diagram with mean segmental values for each parameter [20, 21, 23] and group (i.e. healthy, CAM, LV-NC) was used to obtain an overview of the myocardial mechanics for the entire LV. LV-EF was calculated applying the Simpson’s rule in a biplane technique. LV-mass was evaluated twice at end-diastole: using the area-length method and a triple-plane approach by averaging the masses of the three apical standard views (i.e. 2-, 4-chamber, apical long axis) [33, 34] to better cover all LV regions. The LV-mass of each view was calculated by subtracting the endocardial (LV-EDV_endo_) from the epicardial volume (LV-EDV_epi_) and applying the formula: 1.05 (LV-EDV_epi_–LV-EDV_endo_) [32].

Relative apical systolic longitudinal strain (apical sparing) [26], defined as mean apical systolic longitudinal peak strain/(mean basal peak strain + mean mid-ventricular peak strain), and the absolute value of the ratio of LV-EF/peak global systolic longitudinal strain (EF/S) [24] were evaluated.

Among others Becker et al. [35] and Cheng et al. [36] reported that STE is highly reproducible and minimally affected by intra- and inter-observer variability. To exclude inter-observer variability, measurements were performed by the same experienced investigator (intraobserver variability for longitudinal strain: mean absolute difference 0.23 ± 0.19%, mean relative differences 3.1 ± 2.9%, and coefficient of variation 2.5%).

The variability of STE analysis concerning vendor dependency and independent analyzing software in adults was reported by Risum et al. [37]. In the present study, an influence due to different vendors and calculation algorithms was excluded because the equipment, settings, and analyzing software remained unchanged throughout the study. No temporal trends of the measured parameters due to technical equipment issues were detected.

### Histological analyses of myocardial biopsies

CAM was diagnosed primarily by histology including Congo red-stained right-ventricular myocardial biopsies [38] followed by immuno-histological sub-typing of the amyloid [39] according to the interdisciplinary guidelines for diagnosis and therapy of extra-cerebral amyloidosis issued by the German Society of Amyloid Diseases [40].

### Statistical analysis

A FileMaker database (version 16.0.5.5, FileMaker Inc., Santa Clara/USA) was developed to store echocardiographical data for basic statistics, and to generate polar-diagrams of the final results. Additional statistical analyses were performed using StatView 5.0 (SAS Institute, Cary/USA). Data are presented as mean values ± standard deviation (SD). The *t* test was applied to compare normally distributed values, the Mann–Whitney *U* test for non-normally distributed samples. A *p* ≤ 0.05 was considered statistically significant. The 95% probability threshold for normal values was calculated by applying the two-sigma rule (mean normal value ± 2 SD = 2σ threshold) [20].

## Results

### Conventional echocardiographical parameters (Table 1, upper part)

**Table 1:** Echocardiographic standard parameters and global myocardial longitudinal LV mechanics derived from speckle-tracking echocardiography in individuals with healthy hearts (healthy), cardiac amyloidosis (CAM all), AL-amyloidosis (AL-CAM), ATTR-amyloidosis (ATTR-CAM), and symptomatic LV non-compaction cardiomyopathy (NC)

Parameter	Healthy *n* = 150	CAM all *n* = 59	ATTR-CAM *n* = 29	AL-CAM *n* = 30	NC *n* = 30	*p* value
Left atrial volume index (mm/m^2^)	22.5 ± 4.6	45 ± 13	48 ± 14	42 ± 12	52 ± 18	**Healthy vs CAM: < 0.0001 ** AL- vs ATTR-CAM:0.082 **healthy vs NC: < 0.000 ** **CAM vs NC: 0.0385**
LV-EDD (mm)	50 ± 5	45 ± 7	46 ± 7	45 ± 7	62 ± 9	**Healthy vs CAM: < 0.0001 ** AL- vs ATTR-CAM:0.5854 **healthy vs NC: < 0.0001 ** **CAM vs NC: < 0.0001**
LV-ESD(mm)	31 ± 4	31 ± 8	32 ± 8	30 ± 7	50 ± 12	Healthy vs CAM: 1.0 AL- vs ATTR-CAM:0.3107 **healthy vs NC: < 0.000 ** **CAM vs NC: < 0.0001**
LV-EDV (ml)	89 ± 33	64 ± 29	72 ± 29	57 ± 29	142 ± 57	**Healthy vs CAM < 0.0001 ** AL- vs ATTR-CAM:0.0518 **healthy vs NC: < 0.0001 ** **CAM vs NC: < 0.0001**
LV-ESV (ml)	34 ± 15	31 ± 19	34 ± 17	29 ± 20	100 ± 54	Healthy vs CAM: 0.2301 **AL- vs ATTR-CAM:0.036 ** **healthy vs NC: < 0.0001 ** **CAM vs NC: < 0.0001**
LV-EF (%)	63 ± 5	52 ± 11	53 ± 10	52 ± 11	34 ± 15	**Healthy vs CAM: < 0.0001** AL- vs ATTR-CAM:0.7165 **healthy vs NC: < 0.0001 ** **CAM vs NC: < 0.0001**
E/A	1.7 ± 0.52	2.4 ± 1.7 (*n* = 40/59 with SR)	2.4 ± 1.44 (*n* = 16/29 with SR)	2.5 ± 1.83 (*n* = 24/30 with SR)	1.6 ± 0.8	**Healthy vs CAM: < 0.0001 ** AL- vs ATTR-CAM: 0.855 healthy vs NC: 0.386 **CAM vs NC: 0.0168**
LV-E/e´	6.6 ± 1.57	21.0 ± 7.19	20.4 ± 6.29	21.5 ± 8.03	14.7 ± 7.3	**Healthy vs CAM: < 0.0001 ** AL- vs ATTR-CAM:0.561 **healthy vs NC: < 0.0001 ** **CAM vs NC: 0.0002**
Body surface area (m^2^)	1.87 ± 0.24	1.90 ± 0.20	1.90 ± 0.1	1.90 ± 0.2	1.80 ± 0.24	
Heart rate b/min	64.4 ± 11	72.8 ± 13.7	67.2 ± 12.5	78.3 ± 12.8	79.6 ± 16.8	
LV mass index area length (g/m^2^)	65 ± 14	143 ± 38	149 ± 40	137 ± 35	144 ± 51	**Healthy vs CAM: < 0.0001 ** AL- vs ATTR-CAM:0.2247 **healthy vs NC: < 0.0001 ** CAM vs NC: 0.917
LV mass index triple plane (g/m^2^)	64 ± 17	124 ± 32	134 ± 33	114 ± 28	115 ± 34	**Healthy vs CAM: < 0.0001 ** **AL- vs ATTR-CAM:0.0148** ** healthy vs NC: < 0.0001 ** CAM vs NC: 0.2227
Peak systolic longitudinal strain (%)	− 21.1 ± 3.2	− 10.8 ± 4.59	− 11.5 ± 4.62	− 10.0 ± 4.4	− 8.8 ± 6.5	**Healthy vs CAM: < 0.0001 ** AL- vs ATTR-CAM: 0.208 **healthy vs NC: < 0.0001 ** CAM vs NC: 0.0962
Peak systolic longitudinal strain rate (%/s)	− 1.23 ± 0.31	− 0.75 ± 0.34	− 0.76 ± 0.33	− 0.75 ± 0.34	− 0.64 ± 0.32	**Healthy vs CAM: < 0.0001 ** AL- vs ATTR-CAM: 0.909 **healthy vs NC: < 0.0001 ** CAM vs NC: 0.145
Time-to-peak syst. longitudinal strain (ms)	371 ± 42	350 ± 70	371 ± 66	330 ± 68	389 ± 74	**Healthy vs CAM: 0.0085 ** ** AL- vs ATTR-CAM:0.0223** healthy vs. NC: 0.0661 **CAM vs NC: 0.0168**
Time-to-peak syst. longitudinal strain rate (ms)	181 ± 47	155 ± 63	170 ± 55	139 ± 60	200 ± 83	**Healthy vs CAM: 0.0013 ** **AL- vs ATTR-CAM:0.0433 ** healthy vs. NC: 0.0831 **CAM vs NC: 0.0054**
Pre-systolic stretch index (%)	1.3 ± 2.8	4.8 ± 12.5	5.1 ± 10.9	4.6 ± 12.5	13.2 ± 26.3	**Healthy vs CAM: 0.0014 ** AL- vs ATTR-CAM: 0.871 **healthy vs NC: < 0.0001 ** **CAM vs NC: 0.0436**
Post-systolic index (%)	2.5 ± 3.1	6.0 ± 8.7	5.7 ± 7.8	6.2 ± 9.3	15.9 ± 20.7	**Healthy vs CAM: < 0.0001 ** AL- vs ATTR-CAM: 0.824 **healthy vs NC: < 0.0001 ** **CAM vs NC: 0.0021**
Peak longitudinal displacement (mm)	12.1 ± 2.5	7.3 ± 3.2	8.1 ± 3.1	6.6 ± 3.0	5.6 ± 3.8	**Healthy vs CAM: < 0.0001 ** AL- vs ATTR-CAM: 0.064 **healthy vs NC: < 0.0001 ** **CAM vs NC: 0.0289**
EF/S	3.0 ± 0.5	4.9 ± 1.3	4.6 ± 1.1	5.2 ± 1.5	4.23 ± 1.9	**Healthy vs CAM: < 0.0001 ** AL- vs ATTR-CAM: 0.0860 **healthy vs NC: < 0.0001 ** CAM vs NC: 0.0535
Relative apical longitudinal strain	0.66 ± 0.09	1.06 ± 0.27	1.15 ± 0.30	0.98 ± 0.22	0.73 ± 0.44	**Healthy vs CAM: < 0.0001 ** **AL- vs ATTR-CAM:0.0158 ** healthy vs NC: 0.0755 **CAM vs NC: < 0.0001**
Mechanical dispersion (ms)	32 ± 8	54 ± 18	59 ± 17	50 ± 18	62 ± 22	**Healthy vs CAM: < 0.0001 ** AL- vs ATTR-CAM: 0.0533 **healthy vs NC: < 0.0001** CAM vs NC: 0.0697

*E/A* rate of early (E) to late (A) diastolic LV peak values in the Doppler signal, *E/e*` rate of E to tissue Doppler early diastolic inflow peak value (*e*`), *LV-EDD and LV-ESD* LV end-diastolic and end-systolic diameter, *LV-EDV and LV-ESV*  LV end-diastolic and end-systolic volume, *LV-EF* LV ejection fraction, *EF/S* absolute value of the ratio of LV-EF to global peak strain. Values are mean ± standard deviation. Significant *p* values in bold, not significant in grey

Compared with healthy individuals, patients with CAM had significantly larger left atria (LA), increased LV mass-indices, and ratios of diastolic filling parameters (i.e. E/e´, E/A), whereas LV-EF, end-diastolic LV diameters, and LV volumes were reduced. End-systolic LV diameters and volumes were similar. Patients with AL-CAM had significantly smaller end-systolic and end-diastolic LV-volumes as well as LV mass-indices (triple plane method) compared to individuals with ATTR-CAM.

Data from patients with CAM and LV-NC were similar, except for significantly increased LV diameters and volumes, reduced LV-EF in the LV-NC group, and in parts differing diastolic parameters.

### Global systolic longitudinal myocardial LV mechanics (Table 1, lower part)

Patients with CAM showed increased EF/S compared to healthy individuals and even to patients with LV-NC, no significant difference was found between AL-CAM and ATTR-CAM.

Compared to individuals with healthy heart, patients with CAM and LV-NC demonstrated significantly reduced global systolic peak strain, strain-rate, and displacement, whereas pre-systolic stretch-index and post-systolic index were increased. Time-to-peak systolic strain and time-to-peak systolic strain-rate were reduced in patients with CAM compared to healthy hearts. Contrary to that in LV-NC the values were increased, at least in the basal and mid-ventricular section. In AL-CAM significantly lower values of both time-to-peak strain and time-to-peak strain-rate were found compared to the data in ATTR-CAM.

### Segmental systolic longitudinal myocardial LV mechanics

**Fig. 1:**
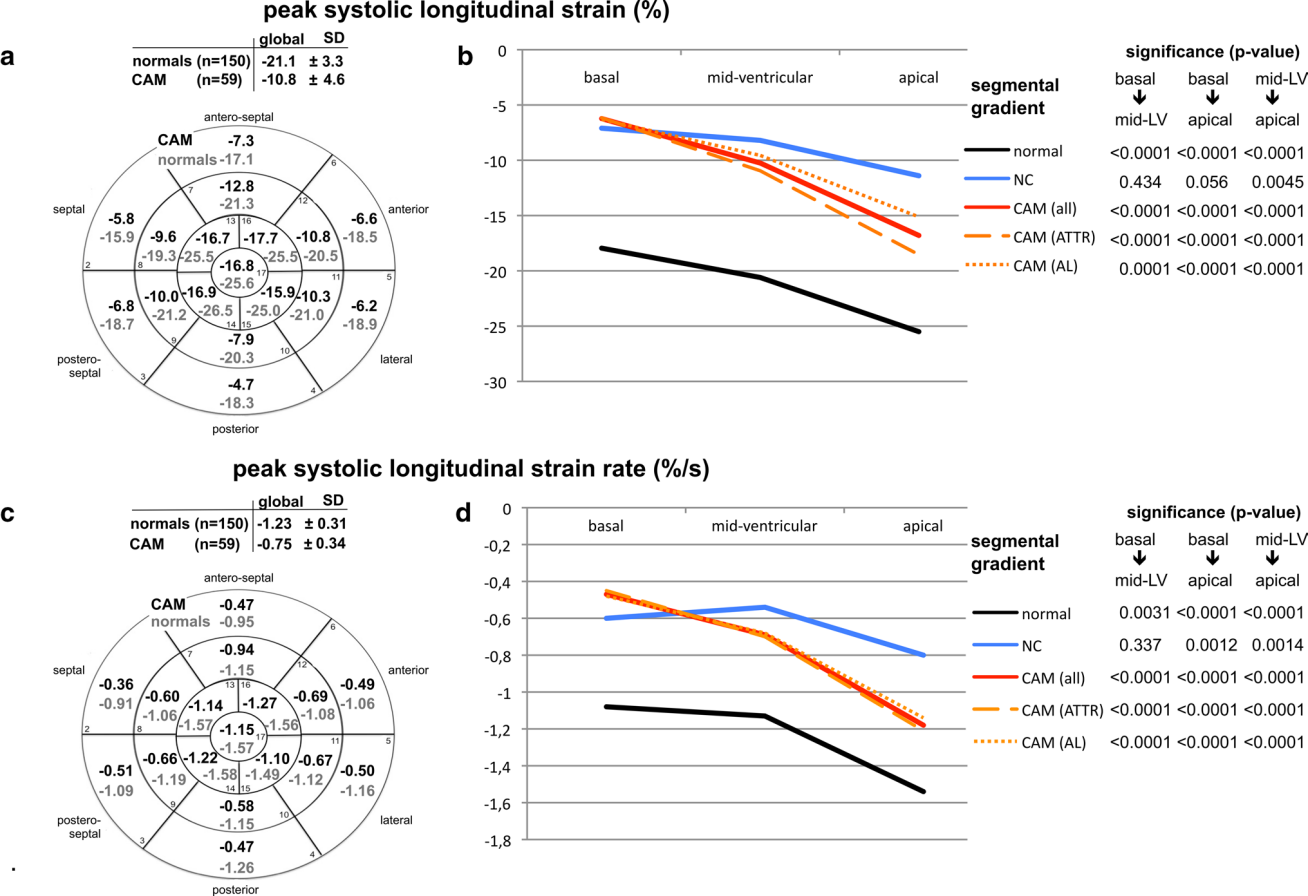
17-segment polar-diagrams of the left ventricle (LV) with mean global and segmental myocardial values of peak systolic longitudinal strain (**a**) and strain-rate (**c**) in cardiac amyloidosis (CAM) versus individuals with healthy hearts (normals). Corresponding graphs (**b**, **d)** demonstrate intraventricular parameter gradients in baso-apical direction relating to the cohort (i.e. normals, CAM all, ATTR-CAM only, AL-CAM only, LV-NC) and LV section (i.e. basal, mid-ventricular, apical). Significance values for differences between LV sections are given on the right and in Table 2. *SD* standard deviation, *CAM (all)* all patients with cardiac amyloidosis, *CAM (ATTR)* patients with subtype ATTR-, *CAM (AL)* patients with subtype AL-amyloidosis, *NC* with symptomatic LV non-compaction cardiomyopathy

**Fig. 2:**
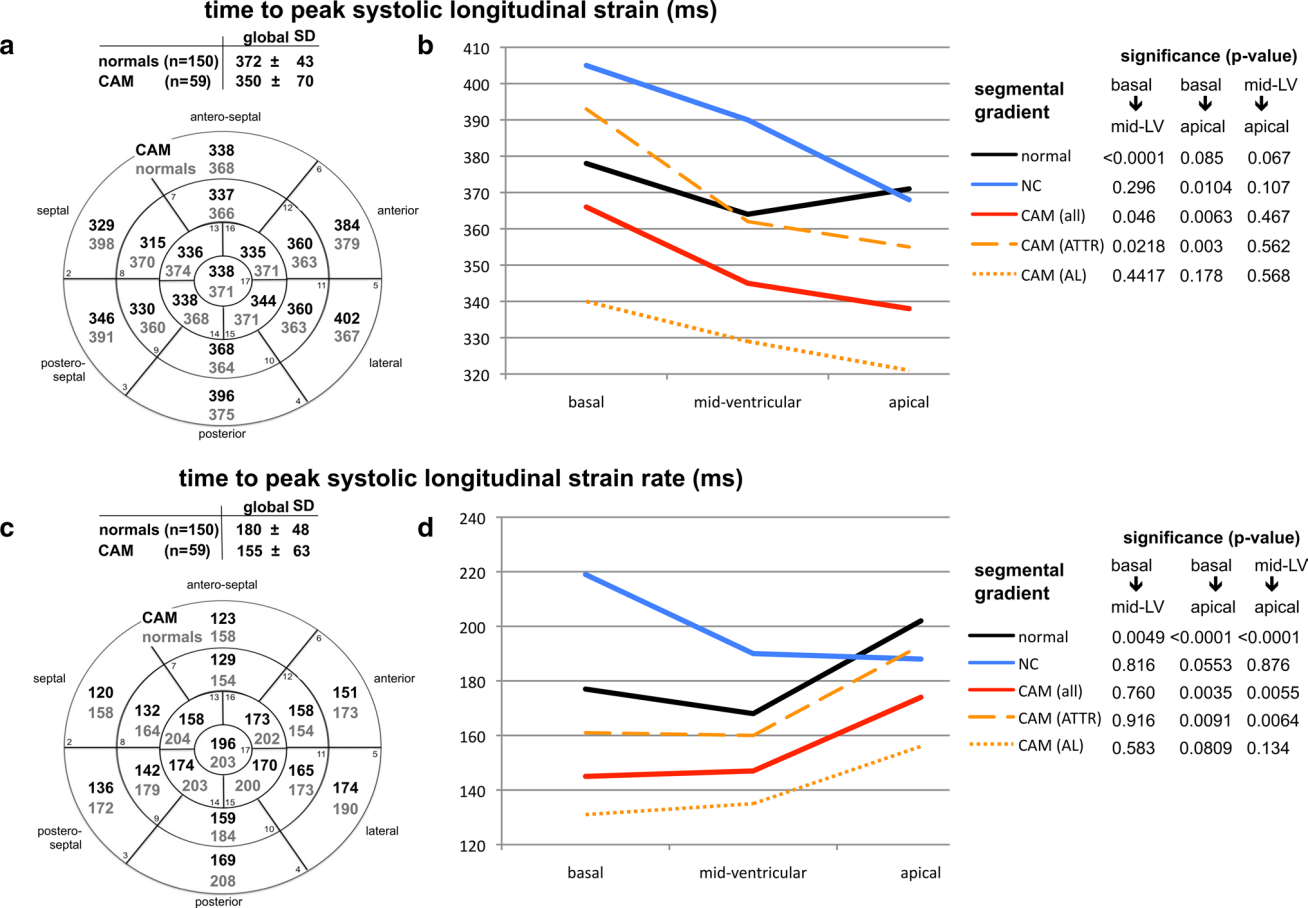
Diagram analogous to Fig. 1: analyses of time-to-peak longitudinal strain (**a**, **b**) and time-to-peak longitudinal strain-rate (**c**, **d**)

**Fig. 3:**
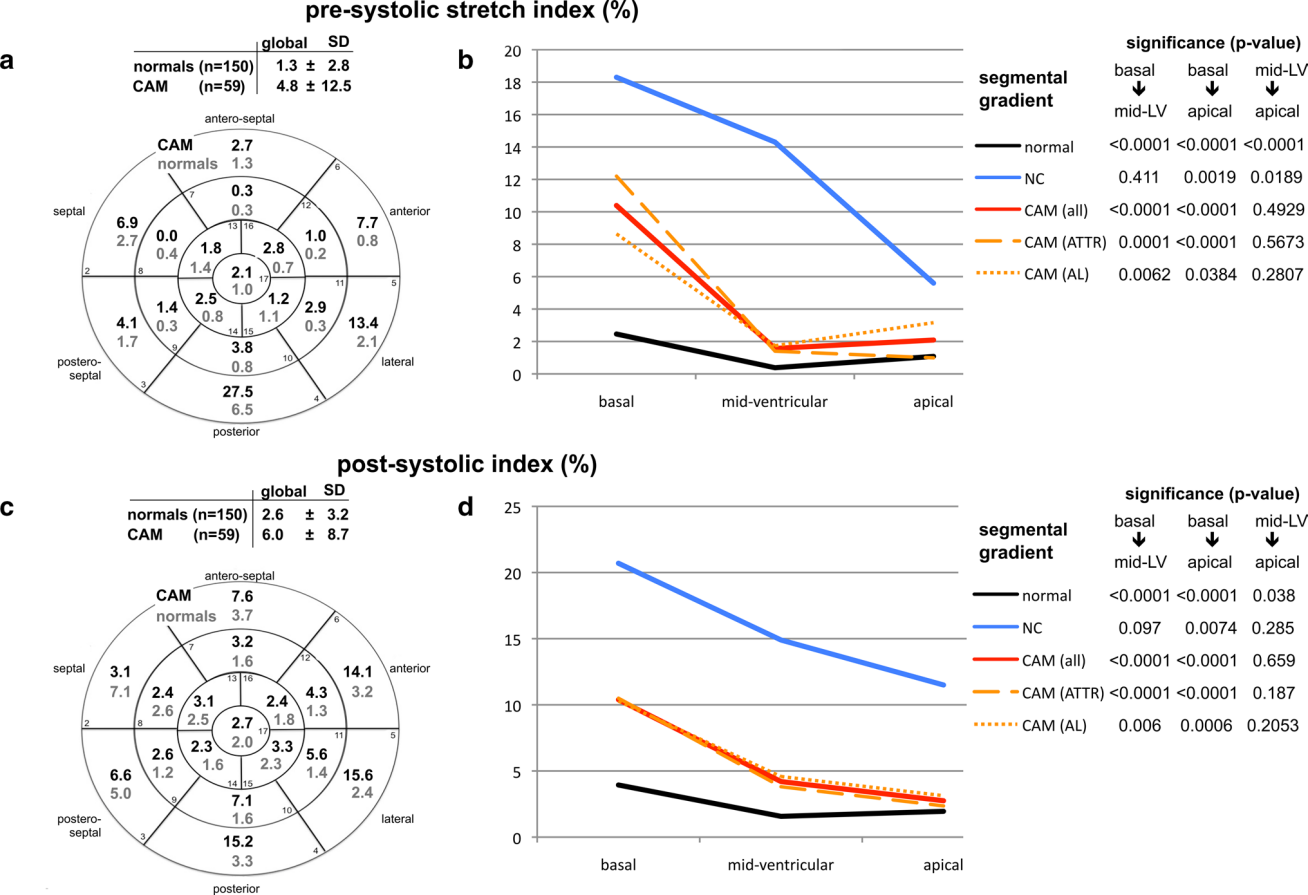
Diagram analogous to Fig. 1: analyses of pre-systolic stretch index (**a**, **b**) and post-systolic index (**c**, **d**)

**Fig. 4:**
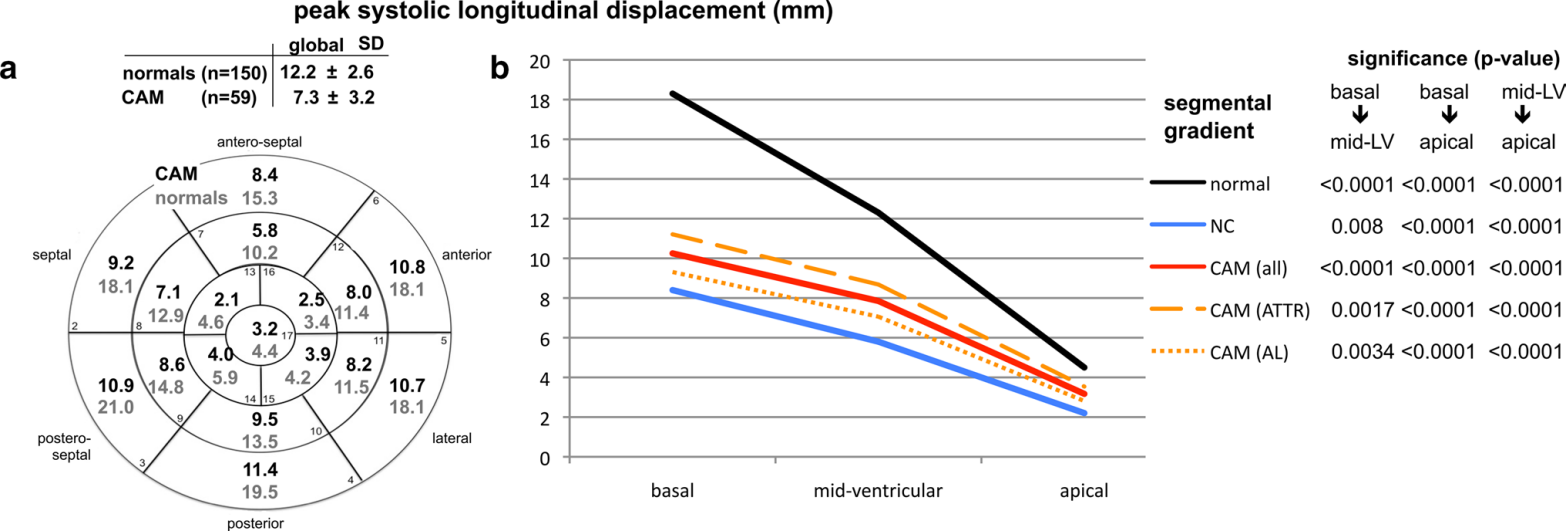
Diagram analogous to Fig. 1: analyses of peak longitudinal displacement

Using polar-diagrams, STE-derived segmental data enabled mappings of the entire LV for each parameter. All significant differences in global STE-derived parameters between healthy hearts, CAM, and LV-NC were also seen in all three LV sections (basal, mid-ventricular, apical) (Figs. 1–4, Table 2) except in peak systolic strain (Fig. 1b) by time-to-peak systolic strain-rate (Fig. 2d); and displacement (Fig. 4b). These parameters showed significant differences between CAM and LV-NC in two sections.

**Table 2:** Comparison of the data for LV longitudinal myocardial mechanics considering the three LV sections: significance values for differences between all individuals with cardiac amyloidosis (CAM all), AL-amyloidosis (AL-CAM), ATTR-amyloidosis (ATTR-CAM), healthy hearts (healthy), and symptomatic LV non-compaction cardiomyopathy (NC) (Figs. 1–4)

Parameter	Comparison CAM all *n* = 59 ATTR-CAM *n* = 29 AL-CAM *n* = 30 Healthy *n* = 150 NC *n* = 30	*p* value		
		Basal	Mid-ventricular	Apical
Peak systolic longitudinal strain	CAM all vs healthy	** < 0.0001**	** < 0.0001**	** < 0.0001**
	ATTR-CAM vs healthy	** < 0.0001**	** < 0.0001**	** < 0.0001**
	AL-CAM vs healthy	** < 0.0001**	** < 0.0001**	** < 0.0001**
	CAM all vs NC	0.3060	**0.0303**	**0.0001**
	ATTR-CAM vs NC	0.3930	**0.0255**	** < 0.0001**
	AL-CAM vs NC	0.4610	0.2581	**0.0161**
	ATTR-CAM vs AL-CAM	0.8440	0.0943	**0.0166**
Peak systolic longitudinal strain-rate	CAM all vs healthy	** < 0.0001**	** < 0.0001**	** < 0.0001**
	ATTR-CAM vs healthy	** < 0.0001**	** < 0.0001**	** < 0.0001**
	AL-CAM vs healthy	** < 0.0001**	** < 0.0001**	** < 0.0001**
	CAM all vs NC	**0.0031**	**0.0032**	**0.0001**
	ATTR-CAM vs NC	**0.0077**	**0.0115**	**< 0.0001**
	AL-CAM vs NC	**0.0237**	**0.0132**	**0.0043**
	ATTR-CAM vs AL-CAM	0.4750	0.7110	0.5376
Time-to-peak longitudinal strain	CAM all vs healthy	0.0684	**0.0013**	** < 0.0001**
	ATTR-CAM vs healthy	**0.0494**	0.7684	**0.0345**
	AL-CAM vs healthy	** < 0.0001**	** < 0.0001**	** < 0.0001**
	CAM all vs NC	**0.0037**	**0.0003**	**0.0094**
	ATTR-CAM vs NC	0.3982	**0.0397**	0.2906
	AL-CAM vs NC	** < 0.0001**	**0.0001**	**0.0009**
	ATTR-CAM vs AL-CAM	**0.0003**	**0.0183**	**0.0085**
Time-to-peak longitudinal strain-rate	CAM all vs healthy	** < 0.0001**	** < 0.0001**	**0.0001**
	ATTR-CAM vs healthy	**0.0068**	0.1788	0.2659
	AL-CAM vs healthy	** < 0.0001**	** < 0.0001**	** < 0.0001**
	CAM all vs NC	** < 0.0001**	** < 0.0001**	0.2978
	ATTR-CAM vs NC	**0.0003**	**0.0121**	0.6997
	AL-CAM vs NC	** < 0.0001**	** < 0.0001**	**0.0440**
	ATTR-CAM vs AL-CAM	**0.0011**	**0.0028**	**0.0265**
Pre-stretch index	CAM all vs healthy	** < 0.0001**	**0.0001**	**0.0216**
	ATTR-CAM vs healthy	** < 0.0001**	**0.0010**	0.7958
	AL-CAM vs healthy	** < 0.0001**	**0.0001**	**0.0004**
	CAM all vs NC	**0.0259**	** < 0.0001**	**0.0134**
	ATTR-CAM vs NC	0.1695	**0.0003**	**0.0060**
	AL-CAM vs NC	**0.0275**	**0.0004**	0.2068
	ATTR-CAM vs AL-CAM	0.3003	0.7116	0.0723
Post-systolic index	CAM all vs healthy	** < 0.0001**	** < 0.0001**	0.0682
	ATTR-CAM vs healthy	** < 0.0001**	** < 0.0001**	0.4294
	AL-CAM vs healthy	** < 0.0001**	** < 0.0001**	**0.0104**
	CAM all vs NC	**0.0001**	** < 0.0001**	** < 0.0001**
	ATTR-CAM vs NC	**0.0007**	** < 0.0001**	**0.0003**
	AL-CAM vs NC	**0.0015**	**0.0001**	**0.0005**
	ATTR-CAM vs AL-CAM	0.9201	0.4647	0.5113
Peak longitudinal displacement	CAM all vs healthy	** < 0.0001**	** < 0.0001**	** < 0.0001**
	ATTR-CAM vs healthy	** < 0.0001**	** < 0.0001**	** < 0.0001**
	AL-CAM vs healthy	** < 0.0001**	** < 0.0001**	** < 0.0001**
	CAM all vs NC	**0.0068**	**0.0010**	0.5377
	ATTR-CAM vs NC	**0.0068**	**0.0001**	0.5518
	AL-CAM vs NC	0.3573	0.0738	0.7825
	ATTR-CAM vs AL-CAM	**0.0291**	**0.0144**	**0.0131**

Significant *p* values in bold, not significant in grey

Between AL- and ATTR-CAM peak displacement (Fig. 4b, Table 2), time-to-peak systolic strain, and time-to-peak systolic strain-rate (Fig. 2b + d, Table 2) differed significantly in all three sections, whereas peak syst. strain varied significantly only in the apical region (Fig. 1b, Table 2).

### Intraventricular baso-apical gradients of longitudinal myocardial mechanics

In healthy hearts LV longitudinal myocardial mechanics showed intraventricular gradients in baso-apical direction (Figs. 1–4 b + d) [20]: absolute values increased from basal to apical for peak strain, strain-rate, and time-to-peak strain-rate, but decrease for pre-systolic stretch-index, post-systolic index, displacement, and time-to-peak systolic strain.

In all disease groups (ATTR-CAM, AL-CAM, LV-NC) significant baso-apical gradients were also found for peak systolic strain (Fig. 1a + b), strain-rate (Fig. 1c + d), pre-systolic stretch-index (Fig. 3a + b), post-systolic index (Fig. 3c + d), and systolic displacement (Fig. 4a + b). Significant intraventricular gradients for time-to-peak systolic strain (Fig. 2b) and time-to-peak systolic strain-rate (Fig. 2d) were present only partially in the various groups (Table 2).

Although the levels of the segmental data were different between the groups, the intraventricular rates of baso-apical changing of the parameters were comparable, except for pre-systolic stretch-index (Fig. 3b), time-to-peak systolic longitudinal strain, and time-to-peak strain-rate (Fig. 2b, d).

### Intraventricular circumferential gradients of the longitudinal myocardial mechanics

In parts the investigated parameters of longitudinal myocardial mechanics showed gradients also in circumferential direction (Figs. 1–4, a + c). In healthy individuals and patients with LV-NC [21], peak longitudinal systolic strain was lowest antero-septally (Fig. 1a), whereas in CAM, a circumferential shift was detected with lowest values posteriorly and highest antero-septally. There was no significant circumferential gradient in peak systolic strain-rate in any of the groups (Fig. 1c) [21]. Pre-systolic stretch-indices decreased septally and increased postero-laterally in CAM and individuals with healthy heart (Fig. 3b). On the contrary, in LV-NC highest values were found septally [21]. The maximum of post-systolic index shifted from the free LV wall (in CAM) to the septal area (healthy + LV-NC) [21] (Fig. 3c), whereas the minimum was detected septally in CAM and postero-laterally in healthy hearts and LV-NC. Concerning time-to-peak systolic strain (Fig. 2a) and time-to-peak strain-rate (Fig. 2c), no definite circumferential gradient was found in healthy hearts or patients with LV-NC. In CAM, gradients were detected from postero-lateral (maximum) to septal (minimum). All three groups (healthy, LV-NC, CAM) showed circumferential gradients of peak systolic displacement with a minimum antero-septally and a maximum posterior to postero-septally (Fig. 4a).

### Relative apical longitudinal systolic strain (Table 1)

The relative apical strain in patients with CAM was significantly increased than that in individuals with healthy-hearts and LV-NC. The data of the LV-NC group and heart-healthy individuals differed only by tendency. In the group with ATTR-CAM this parameter was significantly higher than in the AL-CAM cohort.

## Discussion

Certain STE-derived echocardiographic parameters may help to differentiate various diseases leading to LV myocardial hypertrophy in combination with LV dysfunction [24]. We chose a comparison between CAM versus LV-NC because both are primary myocardial diseases with increased myocardial thickness and LV-mass in combination with severe systolic and diastolic LV dysfunction in contrast to HCM or myocardial adaptations (e.g. due to hypertension, aortic stenosis, etc.) [21, 25, 27–31].

However, the cause of myocardial dysfunction is completely different in Cam versus LV-NC: in CAM the increased myocardial thickness including infiltrated fibrils, in LV-NC the thickened non-compacted, myocardium and the thin compact wall layer as the key abnormality responsible for LV-function [21].

### The diastolic LV-function

In the present study, diastolic LV-dysfunction was worse in CAM (Table 1) [8] than in LV-NC, but it was similar in ATTR- versus AL-CAM. The reason for a more distinct diastolic failure in CAM may be due to both the progressive infiltration of fibrils and increased myocardial thickness. On the contrary, like systolic LV-function, the diastolic function in LV-NC appears to depend almost exclusively on the thin compact myocardial layer in combination with a quasi-inactive non compacted thick layer as burden material [21].

### The global systolic LV-function using LV-EF

Although LV-EF is only a global and imprecise measure of the complex systolic myocardial function, LV-EF/S was introduced to be a sensitive parameter to distinguish CAM with an increased value around 5.5 ± 1.5 compared to hypertrophic cardiomyopathy (HCM) (3.7 ± 0.5) and hypertensive heart disease (3.2 ± 0.4) [25]. Our results for EF/S confirmed these reported data for the CAM group (Table 1). Given that EF/S values in ATTR- and AL-CAM differ only by tendency, this ratio appeared to be unsuitable to differentiate between both subtypes of CAM in the present study. Interestingly, EF/S values in the CAM group were not only significantly higher compared to healthy hearts, but also to patients with LV-NC although the other systolic findings indicated less severe systolic dysfunction in CAM than in LV-NC [21].

### The global systolic longitudinal myocardial LV mechanics using STE-derived parameters

#### Strain, time-to-peak strain and time-to-peak strain-rate

Strain analyses are crucial for the evaluation of myocardial mechanics like in CAM. In our patients with CAM, global longitudinal systolic strain (Table 1) was comparable to values reported by Pagourelias et al. (− 11.0 ± 4.1%) [24], whereas published data for HCM (− 17.9 ± 2.7%) and hypertensive heart disease (− 19.2 ± 2.3%) [24] were only slightly below our values in the healthy control group (Table 1).

The CAM cohort showed significantly shortened time intervals until the reduced peak of longitudinal strain and strain-rate was reached (Fig. 2b, d, Tables 1, 2), whereas both parameters were significantly longer in LV-NC compared with CAM and even healthy hearts. A plausible explanation for this myocardial behavior in LV-NC may be that fibrosis, which is reported to be common in LV-NC [11–13], combined with a non-compacted, quasi-inactive myocardial layer and lengthening of the compact layer due to a dilated LV may elongate the interval. In CAM, the increased myocardial thickness with infiltrated fibrils also suggests a lengthening of the time intervals. However, a reduction of the time intervals was found instead. It is unclear whether this result is due to increased intramyocardial impulse velocity of the conduction system in CAM or a shortened myocardial reaction rate towards an electrical impulse, or both.

Interestingly, the analyses of time-to-peak systolic longitudinal strain and time-to-peak strain-rate revealed significant differences between ATTR-CAM and AL-CAM. Currently, the cause is unknown but apparently, the underlying pathology that caused shortening of these time intervals in CAM, had an intensified impact in AL-CAM. Therefore, further investigation is required to determine whether one or both parameters may have the potential to differentiate between AL- and ATTR-amyloidosis (Fig. 2b, d).

#### Pre-systolic stretch-index, post-systolic index, and systolic longitudinal displacement

The analyses of pre-systolic stretch-index, post-systolic index, and systolic longitudinal displacement indicated severe LV-dysfunction in CAM and LV-NC (Tables 1, 2). Pre-systolic stretch-index is considered to be a determinant of systolic mechanical synchrony and a precondition for subsequent quick contraction movement [41]. Therefore, with proceeding LV-failure, fiber pre-stretch increases. Post-systolic index determines the degree of post-systolic shortening, which generally may be elevated due to ischemia, LV-hypertrophy, or diastolic dysfunction [42, 43]. In accordance with these facts we found significantly higher values of pre-systolic stretch-index and post-systolic index in our patients with CAM compared to healthy hearts and both parameters were even higher in LV-NC versus CAM (Table 2), indicating more severe LV dysfunction in LV-NC.

Finally, an echocardiographical characterization emerged for both myocardial diseases when comparing CAM, healthy hearts and LV-NC taking all investigated STE-derived parameters into account which were summarized in Tab. 3.

**Table 3:** Comparative characterization of global myocardial longitudinal LV mechanics in patients with cardiac amyloidosis (CAM all) versus symptomatic LV-non compaction cardiomyopathy (LV-NC) in relation to data for heart-healthy individuals

Parameter	Healthy hearts (*n* = 150)	CAM all (*n* = 59)	LV-NC (*n* = 30)	*p* value CAM vs LV-NC
Peak systolic longitudinal strain (%)	Ø	↓↓	↓↓(↓)	0.0962
Peak systolic longitudinal strain rate (%/s)	Ø	↓↓	↓↓(↓)	0.145
Time-to-peak systolic longitudinal strain (ms)	Ø	**↓**	**↑**	**0.0168**
Time-to-peak systolic longitudinal strain rate (ms)	Ø	**↓**	**↑**	**0.0054**
Pre-systolic stretch index (%)	Ø	**↑**	**↑↑↑**	**0.0436**
Post-systolic index (%)	Ø	**↑**	**↑↑↑**	**0.0021**
Peak longitudinal displacement (mm)	Ø	**↓**	**↓↓↓**	**0.0289**

Ø normal value, ↑/↓ mildly, ↑↑/↓↓ moderately, ↑↑↑/↓↓↓ severely increased/decreased speckle-tracking-echocardiography-derived parameters compared with healthy hearts. *p* values in bold = significant, in grey = not significant

### The segmental systolic LV-function using STE-derived parameters of longitudinal myocardial LV mechanics

Segmental analyses of myocardial mechanics provided mappings of the absolute values and intraventricular gradients for each investigated parameter [20, 21] (Figs. 1–4). In healthy hearts segmental parameter values changed systematically from basal to apical and circumferentially, documenting gradients in both directions [20] and demonstrating the three-dimensional myocardial activity. In this context, gradients of the investigated longitudinal parameters in circumferential direction should not be mixed up with circumferential deformation such as circumferential strain.

In all three groups in the present study, the baso-apical intraventricular gradients of myocardial mechanics were detected with various rates of baso-apical change of values and on significantly different levels. This indicates that segmental myocardial function is present with different strength in the three groups.

The observed gradients of longitudinal parameters in circumferential direction were dependent on the investigated myocardial disease. However, no specific circumferential shifting pattern of the parameter’s maxima or minima could be identified for either cardiomyopathy compared with healthy hearts.

### Relative apical longitudinal strain and apical sparing

Higher relative apical strain values may occur in cases with reduced basal and/or midventricular strain for any reason (e.g. infarction, myocarditis, etc.). However, increased relative apical strain (apical sparing) appears to be an accepted indicator for the presence of CAM—with reported values ranging from 0.97 ± 0.3 [24, 25] to 2.0 ± 1.8 [26]. Relative apical strain in our patients with CAM confirmed the data of Phelan et al. [26] and Fikrle et al. [44]. However, by trend, a discrete increase of relative apical strain was also present in LV-NC (Tab. 1), although the mid-ventricular and especially apical regions of the LV myocardium were predominantly affected by non-compaction. Therefore, a reduction of myocardial activity in that region with decreased relative apical strain would be more plausible.

In our study, significantly higher relative apical strain values were found in patients with ATTR-CAM versus AL-CAM. The most plausible explanation may be differing distribution patterns of infiltrative material, taking the published findings in cardiac MRI into account. Duncu et al. [45] and Williams et al. [46] reported increased transmural LGE in ATTR-CAM with a LGE-gradient from high basal to low apical values indicating maximum of fibril infiltration in the basal regions. The consequence is a reduction of myocardial mechanics in basal and midventricular LV segments and, therefore, a high relative apical strain. On the contrary, in AL-CAM only subendocardial LGE is seen more often. The result is a lower strain-gradient between basal and apical segments and, therefore, a lower relative apical strain.

#### The inaccuracy of visual detection of apical sparing

An apical sparing seems to be easily visible using the color-coded polar diagram of a longitudinal systolic peak strain analysis. However, the color coding of the currently available echo-analyzing systems does not take into account the significant physiological intraventricular baso-apical gradients of longitudinal strain in healthy individuals [20]. The current reference for color coding is the global, not the regional (i.e. at least basal, mid-ventricular, apical) value of normal longitudinal strain. Therefore, the seemingly quick recognition of apical sparing using this color coding alone may lead to false interpretations. Until an adapted color coding is available and automatic calculation of the relative apical longitudinal strain is implemented, only the time-consuming, manually performed calculation seems to be a reasonable method to detect apical sparing correctly (Figs. 1–4, b + d).

#### The constancy of inverse proportionality between increasing longitudinal strain and decreasing displacement from basal to apical

**Fig. 5:**
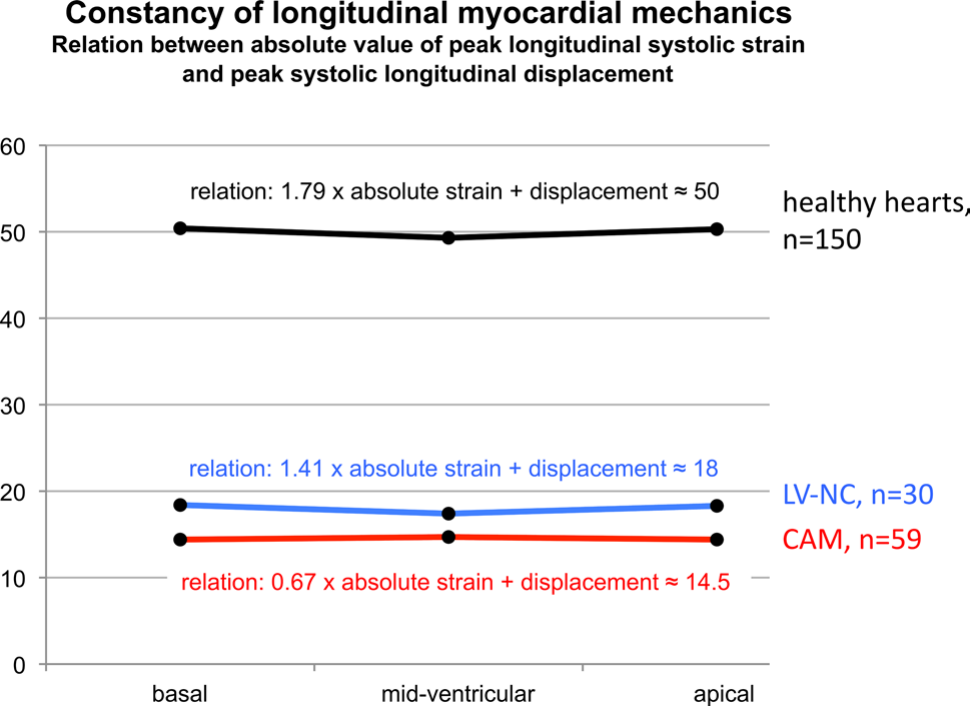
The diagram illustrates the constancy of inverse proportionality between increasing longitudinal strain and decreasing displacement of the left ventricle (LV) from basal to apical including the corresponding mathematical relation. Displayed are the curves of individuals with healthy hearts versus patients with cardiac amyloidosis (CAM) and symptomatic LV non-compaction (LV-NC)

**Fig. 6:**
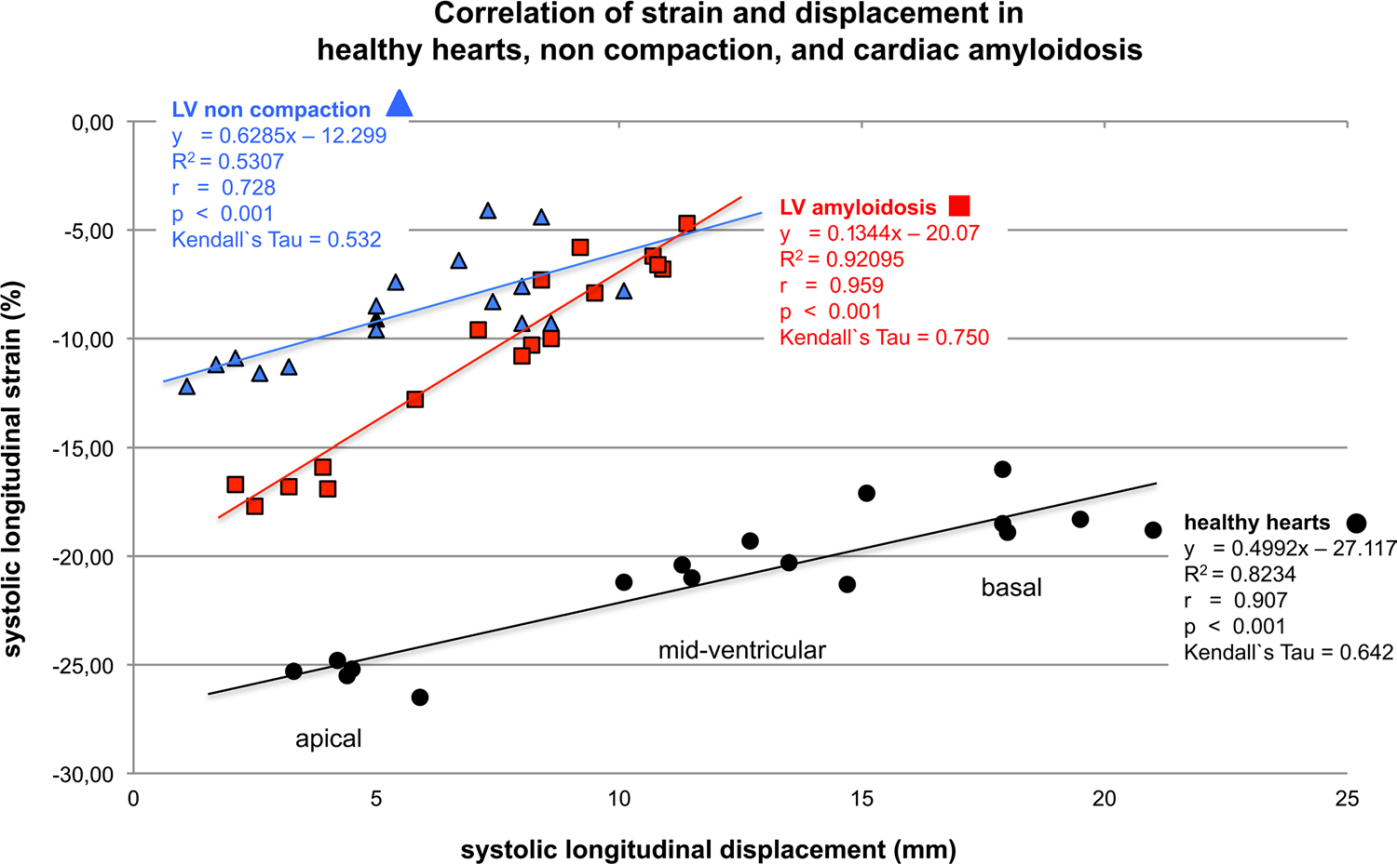
Compared are individuals with healthy hearts (*n* = 150), symptomatic LV non-compaction cardiomyopathy (*n* = 30), and cardiac amyloidosis (*n* = 59) relating to the 17 segments of the polar-diagram (Figs. 1–4). The location of the resulting line of each group within the diagram is dependent on systolic LV-function: lowest line represents healthy individuals, the top line (non-compaction) the worst. The slope of the line is nearly equal for individuals with healthy hearts and LV non-compaction whereas patients with amyloidosis showed a steeper slope indicating an increased difference of strain values between LV apex and basis (apical sparing) in amyloidosis

In healthy hearts, a nearly constant inverse proportionality was observed from basal to apical throughout the entire ventricle between the increasing peak longitudinal systolic strain and the decreasing longitudinal systolic displacement [20] (Fig. 5). This phenomenon was also observed in patients with CAM and LV-NC [21] but with altered mathematical relations due to reduced LV-function. The localization of the resulting line of each group in the diagram (Fig. 6) is dependent on systolic LV function. The most upper position of a graph (LV-NC) represents the worst systolic function between the three groups. The slope of the graphs for healthy hearts and patients with symptomatic LV-NC were comparable whereas patients with CAM showed a steeper curve, due to an increased baso-apical gradient of strain in amyloidosis (apical sparing). Whether these different relationships between both parameters are specific for healthy hearts and both cardiomyopathies, needs a comparison with other cardiomyopathies or adaptive processes of the LV myocardium.

## Clinical application

When investigating myocardial diseases or even myocardial adaptation/remodeling, strain analyses are the adequate evaluation to gain information about global and segmental impact of the disease. In combination with standard echocardiographical parameters the evaluation of strain-rate, time-to-peak strain and strain-rate and the other STE-derived parameters may follow to differentiate between diverse myocardial diseases or adaptations. When interpreting STE analyses baso-apical gradients of longitudinal parameters of myocardial mechanics should kept in mind as physiological, which is especially important in case of relative apical strain.

A kind of constant baso-apical mechanics can be evaluated because strain increases from basal to apical rapidly whereas displacement decreases in a nearly constant inverse proportionality. Cardiomyopathies with reduced systolic function show baso-apical gradients similar to normals but on a reduced level. However, it becomes feasible to differentiate between AL-CAM versus ATTR-CAM.

### Summary of significant differences between AL- and ATTR-amyloidosis

Patients with AL-CAM compared with ATTR-CAM showed significantly lower end-diastolic and end-systolic LV-volumes, LV mass-indices (triple plane technique), time-to-peak systolic longitudinal strain, time-to-peak longitudinal strain-rate, and relative apical longitudinal strain, whereas EF/S was higher.

A lower mass-index in AL-CAM is plausible because end-systolic and end-diastolic LV-volumes were lower than in ATTR-CAM but myocardial thickness was similar in both subgroups. Possible causes for differences in the other parameters may be the lower mean age of patients with AL-CAM compared with ATTR-CAM (79.2 ± 6.6 vs 66.0 ± 8.7), the gender distribution in the subgroups (27 males/2 females vs 19 males/11 females), or different stages of the disease in the two groups. However, another plausible explanation is the different protein-structure and distribution of the fibrils and the reaction of surrounding myocardial tissue, which may cause different myocardial mechanics of AL- and ATTR-CAM on a cellular level.

## Limitations

Our findings need to be interpreted in light of limitations. Age-related differences of especially longitudinal peak strain have been reported to be predominantly present for the apical LV section in healthy hearts when comparing between age-groups of 30–39 and > 60 years [47]. In our study, the mean age of the healthy cohort was 33 years and 49 years in the LV-NC group, suggesting that age should not have had an important impact on the between-group comparison. In contrast, the mean age of the CAM group was approximately 71 years, which may influence the comparison of longitudinal strain between the groups for the apical region.

The conditions of the study were not directly comparable to those of routine daily practice, because we evaluated LV myocardial mechanics for all groups under identical technical conditions in a single center. In addition, our findings cannot be generalized to analyses performed with other types or brands of echocardiographic equipment. Furthermore, patient groups with CAM and LV-NC were selected because patients with bundle branch blocks were not accepted due to a potential effect of these rhythm disorders on STE analyses.

## Conclusion

In patients with myocardial diseases analyses of global and especially segmental myocardial LV mechanics using STE is important. Although CAM and LV-NC showed increased myocardial thickness in combination with severe systolic and diastolic LV-dysfunction, both diseases demonstrated significant differences of global and segmental longitudinal myocardial mechanics of the LV and could be characterized. Differentiation between ATTR-CAM and AL-CAM may become feasible, because several parameters were significantly different between both subtypes of CAM. Whether an echocardiographical characterization for different myocardial diseases or even myocardial adaptive processes using STE-derived parameters is possible, needs investigations of other cardiomyopathies to allow comparisons.
